# An Incidental Finding of Eagle Syndrome Post-Losartan-Induced Angioedema

**DOI:** 10.7759/cureus.39334

**Published:** 2023-05-22

**Authors:** Henry Mann, Sagar Pandey, Sindhu C Pokhriyal, Josef Kusayev, Alix Dufresne

**Affiliations:** 1 Internal Medicine, One Brooklyn Health System Interfaith Medical Center, Brooklyn, USA; 2 Medical School, New York Medical College, Valhalla, USA; 3 Cardiology, One Brooklyn Health System Interfaith Medical Center, Brooklyn, USA

**Keywords:** losartan-induced angioedema, angioedema, arb, radiology, anatomy, cardiology, eagle syndrome

## Abstract

Eagle syndrome is a condition that can present with a wide range of clinical manifestations, including orofacial pain, altered sensation, dysphagia, tinnitus, and ear pain, and is caused by the abnormal elongation of the styloid process or the mineralization of the stylohyoid ligament. We present a case of an incidental finding of Eagle syndrome in a 48-year-old African American patient with losartan-induced angioedema. The patient complained of a foreign body sensation in his throat and mild dysphagia, and a computed tomography scan of his neck showed ossification of bilateral stylohyoid ligaments. This case report highlights the importance of being on the lookout for other pathologies when ordering imaging for primary diagnoses.

## Introduction

Eagle syndrome refers to the symptom complex that develops secondary to abnormal elongation of the styloid process or mineralization of the stylohyoid ligament, which results in the disruption of the normal functioning of the neighboring neurovascular structures. Clinical presentation can include orofacial pain, altered sensation, dysphagia, odynophagia, tinnitus, and ear pain [1,2]. The wide range of presentation has been ascribed to the extent of the abnormal ossification of the abovementioned structures and their relationship with cranial nerves 5, 7, 9, and 10 and the internal carotid artery [1]. Our patient complained of a foreign body sensation in his throat and mild dysphagia. Here, we present a case of an incidental finding of Eagle syndrome in a 48-year-old patient with losartan-induced angioedema requiring tracheostomy.

## Case presentation

**Figure 1:**
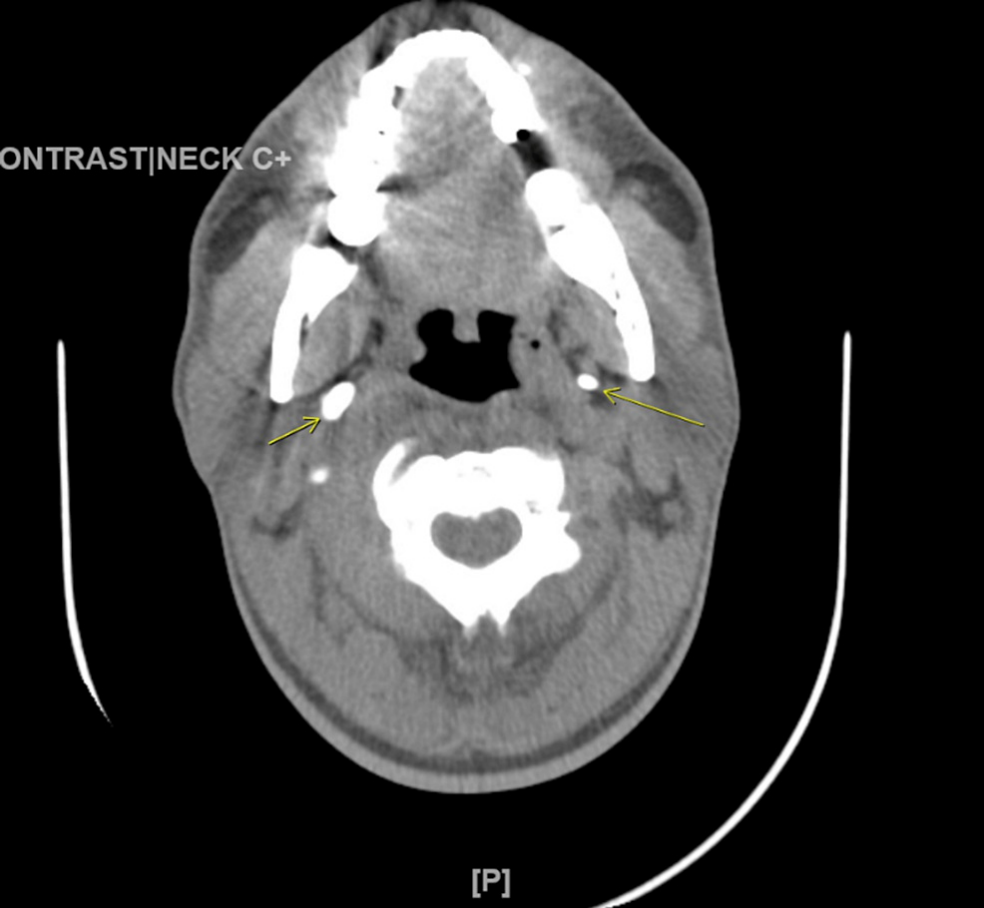
CT neck without contrast before decannulation Transverse view at the C3/C4 vertebra level showing ossification of the bilateral stylohyoid ligaments

Our patient is a 48-year-old African American male with a past medical history of end-stage renal disease on dialysis, acute pancreatitis, diabetes mellitus, and hypertension, who initially presented to the emergency department with complaints of dizziness and bilateral leg edema after missing a hemodialysis session. During the course of his hospitalization, the patient developed hypotension and hoarseness with significant swelling of his face and neck secondary to losartan-induced angioedema; the patient eventually underwent tracheostomy intubation due to difficult oropharyngeal intubation and was started on vasopressors in the intensive care unit. The patient continued to improve on ventilatory support and was extubated a few days later. Computerized tomography (CT) scan of the neck without contrast (Figure 1), done before decannulation of his tracheostomy, showed ossification of approximately 3.2-cm-long bilateral stylohyoid ligaments. After decannulation, the patient initially complained of difficulty swallowing for a few days and failed the initial swallow evaluation. However, he showed subsequent clinical improvement, developing a good gag reflex, and began to tolerate the slow graduated introduction of solid food. Nonetheless, the patient had non-specific complaints of foreign body sensation in his throat and mild dysphagia. Cardiovascular, respiratory, and gastrointestinal physical examinations were normal. Examination of the temporomandibular joint was normal with no tenderness elicited on masticatory movements. The patient’s face was bilaterally symmetrical with no drooping. The oral examination did not reveal a palpable styloid process, visible oral ulcers, or areas of tenderness/induration; reproducible tenderness on palpation of the tonsillar fossa was absent too. The patient was commenced on corticosteroids and was ultimately able to tolerate a complete renal diet after a few weeks; his symptoms of foreign body sensation and dysphagia did not improve totally, however. Unfortunately, the patient experienced a sudden cardiac arrest a few months after this incident of angioedema and died.

## Discussion

Eagle syndrome refers to the symptom complex that develops secondary to abnormal elongation of the styloid process or mineralization of the stylohyoid ligament, which results in the disruption of the normal functioning of the neighboring neurovascular structures. Clinical presentation can include orofacial pain, altered sensation, dysphagia, odynophagia, tinnitus, and ear pain [1,2]. A clinical study comprising 58 symptomatic patients with an elongated styloid process found that vague pain in the neck, foreign body sensation, throat pain, and pain on swallowing were the most common presentations [3]. In our case, the patient had a foreign body sensation in his throat and mild dysphagia. Although post-extubation neuromuscular weakness and trauma could also explain these symptoms, the finding of bilaterally calcified stylohyoid ligaments on the CT neck supported the likelihood of Eagle syndrome. Lack of lancinating and sharp pains in typical anatomical distribution excluded neuralgias as one of the differentials.

Theories behind the development of Eagle syndrome include an embryonic origin from retained cartilage from Reichert’s cartilage, abnormal ossification of the stylohyoid ligament, trauma-induced reactive hyperplasia/metaplasia, anatomic variation of the styloid process, and age-related chronic inflammation [4]. Cases of Eagle syndrome following trauma, radiation, surgery, or tonsillectomy have been reported, but the time interval between this supposed inciting event and the subsequent development of Eagle syndrome was years [5]. Eagle syndrome, in our case, was likely a result of an underlying anatomical aberration, which was visualized as an incidental finding on the CT neck following the development of angioedema.

Eagle syndrome is primarily managed medically with analgesics, corticosteroids, antidepressants, and antiepileptic drugs [3]. Surgical treatment involves styloidectomy and is limited to patients with severe symptoms as well as those unresponsive to medical therapy [6]. Our patient was responsive to corticosteroids, though his symptoms did not completely improve.

## Conclusions

Eagle syndrome should always be kept as a differential in patients presenting with cervicofacial pain with or without associated oropharyngeal symptoms. While history and intraoral palpation of a potential elongated styloid process can aid in the diagnosis, imaging tests, especially CT head and neck, are vital and would show abnormal elongation of the styloid process or mineralization of the stylohyoid ligament. This case report highlights the importance of being on the lookout for other pathologies when ordering imaging for primary diagnoses.
